# SIRT1 and c-Myc Promote Liver Tumor Cell Survival and Predict Poor Survival of Human Hepatocellular Carcinomas

**DOI:** 10.1371/journal.pone.0045119

**Published:** 2012-09-14

**Authors:** Kyu Yun Jang, Sang Jae Noh, Nadja Lehwald, Guo-Zhong Tao, David I. Bellovin, Ho Sung Park, Woo Sung Moon, Dean W. Felsher, Karl G. Sylvester

**Affiliations:** 1 Department of Pathology, Chonbuk National University Medical School and Research Institute of Clinical Medicine, Jeonju, Republic of Korea; 2 Department of Surgery, Division of Pediatric Surgery, Stanford University School of Medicine, Stanford, California, United States of America; 3 Department of General, Visceral and Pediatric Surgery, School of Medicine, Heinrich Heine University, Duesseldorf, Germany; 4 Department of Medicine, Division of Oncology, and Cancer Biology Program, Stanford University School of Medicine, Stanford, California, United States of America; The University of Hong Kong, Hong Kong

## Abstract

The increased expression of SIRT1 has recently been identified in numerous human tumors and a possible correlation with c-Myc oncogene has been proposed. However, it remains unclear whether SIRT1 functions as an oncogene or tumor suppressor. We sought to elucidate the role of SIRT1 in liver cancer under the influence of c-Myc and to determine the prognostic significance of SIRT1 and c-Myc expression in human hepatocellular carcinoma. The effect of either over-expression or knock down of SIRT1 on cell proliferation and survival was evaluated in both mouse and human liver cancer cells. Nicotinamide, an inhibitor of SIRT1, was also evaluated for its effects on liver tumorigenesis. The prognostic significance of the immunohistochemical detection of SIRT1 and c-Myc was evaluated in 154 hepatocellular carcinoma patients. SIRT1 and c-Myc regulate each other via a positive feedback loop and act synergistically to promote hepatocellular proliferation in both mice and human liver tumor cells. Tumor growth was significantly inhibited by nicotinamide *in vivo* and *in vitro*. In human hepatocellular carcinoma, SIRT1 expression positively correlated with c-Myc, Ki67 and p53 expression, as well as high á-fetoprotein level. Moreover, the expression of SIRT1, c-Myc and p53 were independent prognostic indicators of hepatocellular carcinoma. In conclusion, this study demonstrates that SIRT1 expression supports liver tumorigenesis and is closely correlated with oncogenic *c-MYC* expression. In addition, both SIRT1 and c-Myc may be useful prognostic indicators of hepatocellular carcinoma and SIRT1 targeted therapy may be beneficial in the treatment of hepatocellular carcinoma.

## Introduction

Hepatocellular carcinoma (HCC) is the fifth most common cancer and the third most frequent cause of cancer mortality worldwide [1]. Genetic alterations in HCC have been extensively studied yielding the identification of broad molecular categories of HCC [2]. Among numerous potential oncogenic pathways, c-Myc has been observed to be a potent initiating oncogene of liver tumors and inactivation of c-Myc is sufficient to induce sustained regression of MYC-initiated liver tumors in mice [3]. Intriguingly, c-Myc activates the tumor suppressor p53, therefore, additional regulatory mechanisms that are closely related with the oncogenic potential of c-Myc and involve the inactivation of p53 could be essential. Among the direct inhibitors of the p53 protein, SIRT1 is emphasized for its deacetylation activity [4], [5]. In addition, a positive feedback loop between c-Myc and SIRT1 during tumorigenesis would imply a predominant oncogene function for SIRT1 [6], [7]. Conversely, a tumor suppressive role for SIRT1 is suggested by a reciprocal transcriptional control mechanism between c-Myc and SIRT1 [8]. Thus, the role of SIRT1 in human tumors with oncogenic MYC expression remains controversial. Overall, independent of MYC, the deacetylation mediated inhibition of several tumor suppressors including FoxO3 [9], Rb [10], and Ku70 [11], together suggest that SIRT1 has significant tumor promoting activity [12], [13]. Moreover, recent reports have shown that the expression of SIRT1 is associated with a poor prognosis in specific human tumors including hepatocellular carcinoma [14], gastric cancer [15], breast cancer [16], and diffuse large B cell lymphoma [17]. SIRT1 expression has additionally been implicated as a contributing mechanism for increased resistance to anticancer agents [18], [19]. However, there are additional conflicting reports regarding the tumor suppressing capability of SIRT1 [8], [20], [21]. In ovarian cancer patients, SIRT1 expression predicts a favorable prognosis despite high expression in malignant tumors compared with benign or borderline tumors [22]. In colon cancer, SIRT1 was found to negatively regulate the oncoprotein â-catenin [21]. Accordingly, the effect of SIRT1 may vary according to the cell type, stage of tumor development, and accompanying mutation status of tumor related genes.

Despite the prevalence of HCC and its association with c-Myc and SIRT1, there have been few reports describing the biologic role of SIRT1 in liver cancer [14], [23]. Therefore, to investigate the role of SIRT1 in liver cancer and its relationship to c-Myc, we utilized a mouse model of liver tumorigenesis under the genetic control of conditional oncogenic c-MYC. We also extend these studies clinically to a cohort of human HCC tissue.

## Results

### Expression of c-Myc and SIRT1 and the Effect of SIRT1 on Cellular Proliferation in Tet-O-MYC Cell

**Figure 1:**
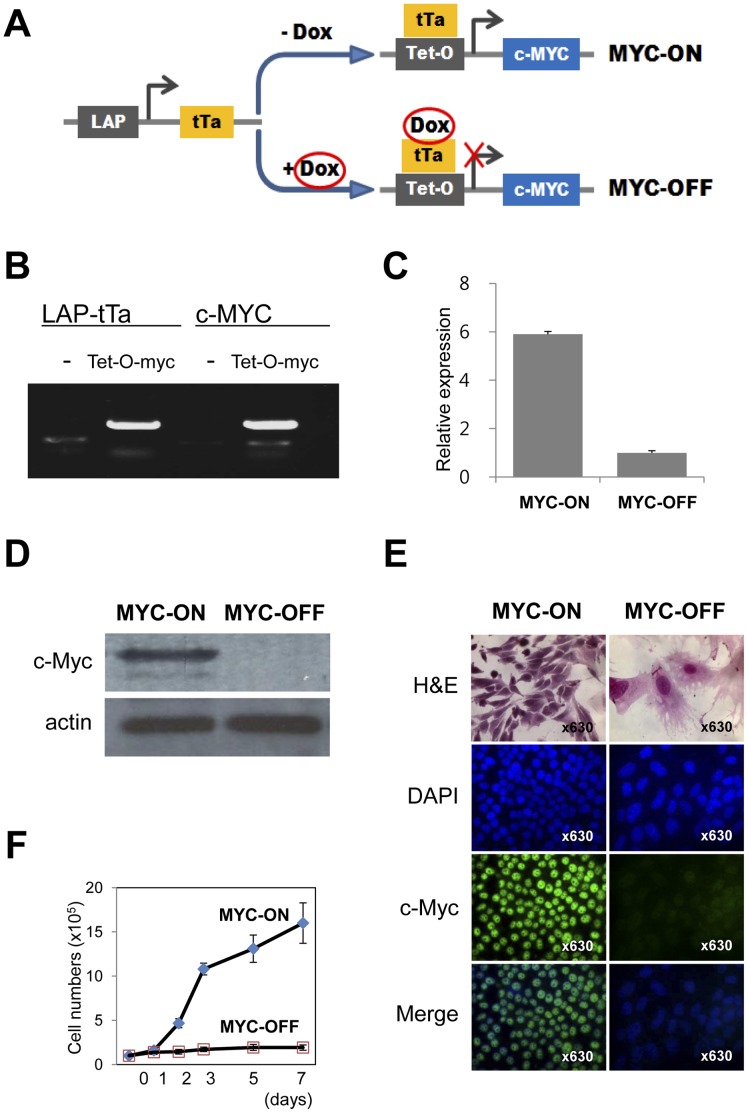
Biological characteristics of a conditional c-Myc mouse liver tumor cell line. A) A tumor cell line derived from the bitransgenic animal containing the liver associated protein (LAP) promoter driving the tetracycline transactivating protein (tTa) and c-Myc under the control of the tetracycline-response promoter (Tet-O). Addition of doxycycline prevents tTa protein from activating the Tet-O promoter. Absence of doxycycline triggers a conformational change that enables Tet-O binding, activation, and c-Myc transcription. B) Polymerase chain reaction indicates that Tet-O-MYC cells are positive for both LAP-tTa and c-Myc transcripts. C) MYC-ON cells show higher expression of messenger RNA for c-Myc than MYC-OFF cells by quantitative real-time polymerase chain reaction. D) Western blot result shows c-Myc expression only in the MYC-ON cells. E) Morphologically, MYC-OFF cells are larger than MYC-ON cells and have larger nuclei, increased cytoplasm, inconspicuous nucleoli, decreased nuclear-cytoplasmic ratio and more cytoplasmic processes. Immunofluoresence staining demonstrates increased c-MYC expression in the nuclei of the MYC-ON cells compared to MYC-OFF cells (original magnification×63). F) The proliferative activity of Tet-O-MYC cells is controlled by c-Myc. Removal of oncogenic c-Myc expression significantly attenuates Tet-O-MYC cell proliferation.

**Figure 2:**
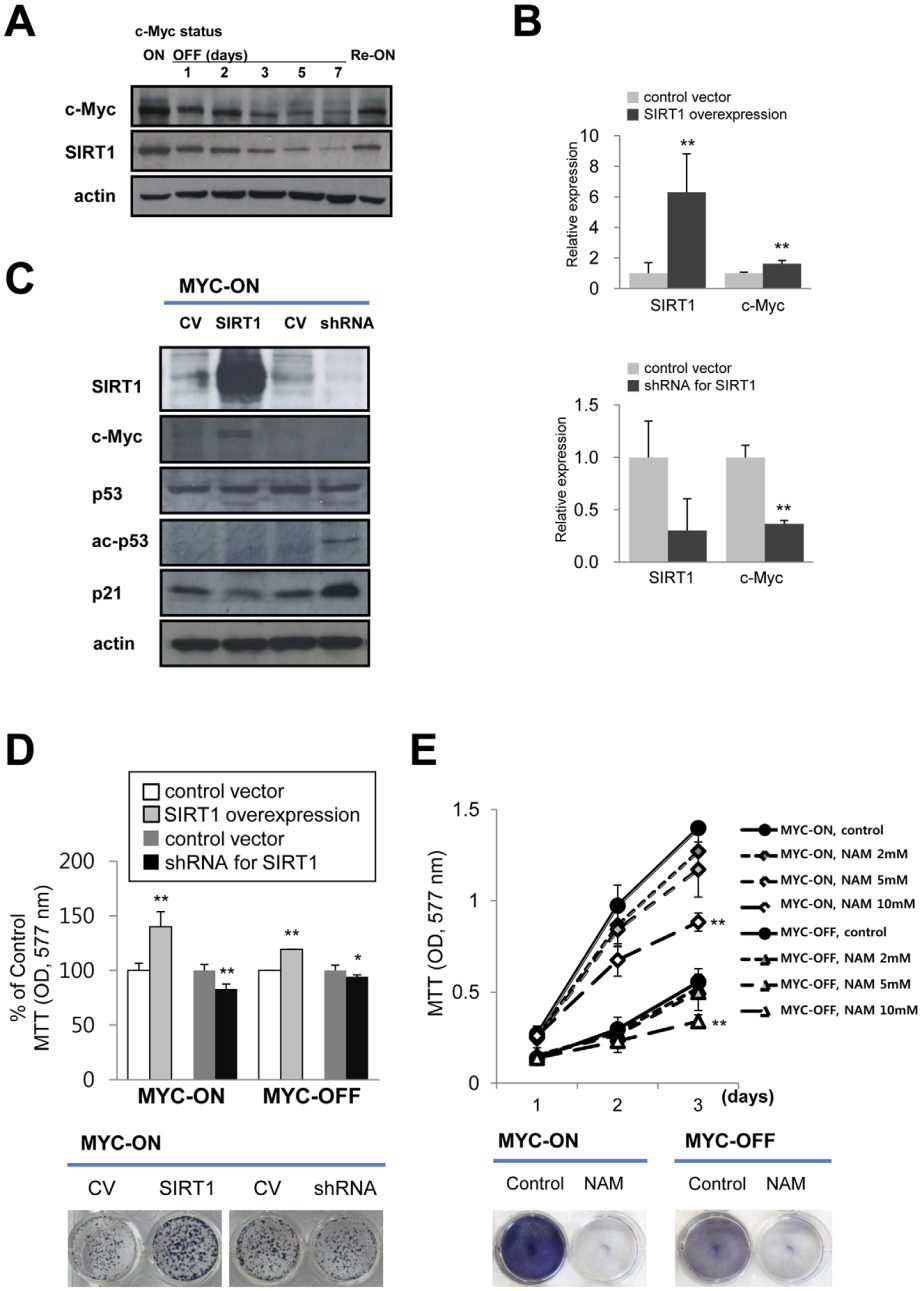
SIRT1 expression correlates with c-Myc expression in Tet-O-MYC cells and influence on proliferation of cells. A) SIRT1 expression directly correlates with c-MYC in a time dependent manner. When oncogenic c-Myc expression is restored over five days (Re-ON), increased SIRT1 expression returns. B and C) Quantitative real time PCR (qPCR) and Western blot. After transfection of Tet-O-MYC cells with a SIRT1 over-expression plasmid, the expression of SIRT1 mRNA and protein as well as c-Myc significantly increases compared with control vector (CV). Knock down of SIRT1 by shRNA against SIRT1 resulted in decreased expression of mRNA and protein for SIRT1 and c-Myc, as well as increased acetylation of p53 protein (ac-p53) and increased expression of p21. D) SIRT1 overexpression promotes increased proliferation. Knock-down of SIRT1 decreased proliferation as demonstrated by MTT and colony forming assay. E) Nicotinamide (NAM), a SIRT1 inhibitor, significantly decreases the proliferation of both MYC-ON and MYC-OFF cells. The MTT assay was performed after seeding of 2×10^3^ cells for two to three days. Colony forming assay were performed in the presence of 10 mM NAM. A colony-forming assay was performed by culture of 2×10^3^ cells for 7 days in 24-well culture plates. A single asterik indicates *P*<0.05, two asteriks indicate *P*<0.001.

In order to investigate the role of SIRT1 in liver tumorigenesis we utilized bitransgenic Tet-O-MYC mice (Tet-O-MYC mice) and primary culture tumor cells (Tet-O-MYC cell) derived from established liver tumors (Figure 1 A and B). Expression of c-Myc protein in Tet-O-MYC cells was successfully controlled by doxycycline. In Tet-O-MYC cells, the addition of 5 ng/ml doxycycline prevents c-Myc transcription (Figure 1 A). MYC-ON cells display increased expression of c-Myc mRNA (Figure 1 C) and c-Myc protein (Figure 1 D) compared to MYC-OFF cells. Morphologically, MYC-OFF cells demonstrate larger nuclei and more abundant cytoplasm than MYC-ON cells. In addition, intranuclear c-Myc expression dramatically decreased in MYC-OFF cells as demonstrated by immunofluoresence staining for c-Myc (Figure 1 E). The proliferative activity of Tet-O-MYC cells was controlled by c-Myc expression. Specifically, when oncogenic c-MYC expression is relieved, a time dependent decrease in cellular proliferation is observed (Figure 1 F). In parallel, the expression of SIRT1 protein strongly correlated with c-MYC expression in a time dependent manner. Moreover, in response to the re-activation of oncogenic c-MYC, the expression of SIRT1 was reversed (Figure 2 A). To further investigate the influence of SIRT1 on the proliferation of liver tumor cells according to c-Myc expression status, a strategy of genetic knock-down or overexpression of SIRT1 in Tet-O-MYC cells was utilized. The overexpression of SIRT1 corresponded to an increased expression of c-Myc mRNA and protein. Conversely, knock down of SIRT1 by shRNA was associated with decreased expression of c-Myc (Figure 2 B and C). In the c-Myc tumor model, significant c-MYC expression is predominantly contributed by the LAP-tTa-Tet-O-MYC system. Therefore, we submit that the demonstrated increase (1.619 times) is significant since SIRT1 contributes to further MYC expression despite a very high expression level induced by the Tet-O-MYC system. Furthermore, SIRT1 knockdown decreased c-Myc expression by ∼65% (Figure 2 B), suggesting that SIRT1 robustly influences c-MYC expression under conditions that are not effected by the Tet-O-MYC system. Together these results demonstrate a direct correlation in expression between SIRT1 and c-Myc. As evidence of the effect of the functional knock-down of SIRT1, a significant increase in acetylated p53 and p21 was also detected (Figure 2 C). The overexpression of SIRT1 induced the proliferation of both MYC-ON and MYC-OFF cells and knock-down of SIRT1 by shRNA resulted in decreased proliferation of both MYC-ON and MYC-OFF cells (Figure 2 D). However, the inhibitory effects on cellular proliferation with knockdown of SIRT1 were marginal on both MYC-ON and MYC-OFF cells. The proliferative activity of MYC-ON cell is mainly contributed by the LAP-tTa-Tet-O-MYC system. Therefore, the effect on cellular proliferation of SIRT1 knockdown appeared marginal. However, a decrease in cellular proliferation by ∼20% suggests a not insignificant biologic effect of SIRT1. In contrast to the marginal effects of SIRT1 knockdown, the proliferation of MYC-OFF cells relates to the very low proliferative potential of MYC-OFF cells as shown in Figure 1 F. Furthermore, in the Tet-O-MYC tumor model, it has been reported that cessation of c-Myc expression is sufficient to induce regression of established tumors *in vivo* [3]. NAM, an established physiologic chemical inhibitor of SIRT1, also significantly decreased the proliferation and colony forming capabilities of both MYC-ON and MYC-OFF cells (Figure 2 E). Together, these results suggest a survival benefit of SIRT1 expression under either genetic or chemical control in liver cells and the corresponding influence of oncogenic c-Myc.

### Effects of SIRT1 on Cellular Proliferation in Human Hepatocellular Carcinoma

**Figure 3:**
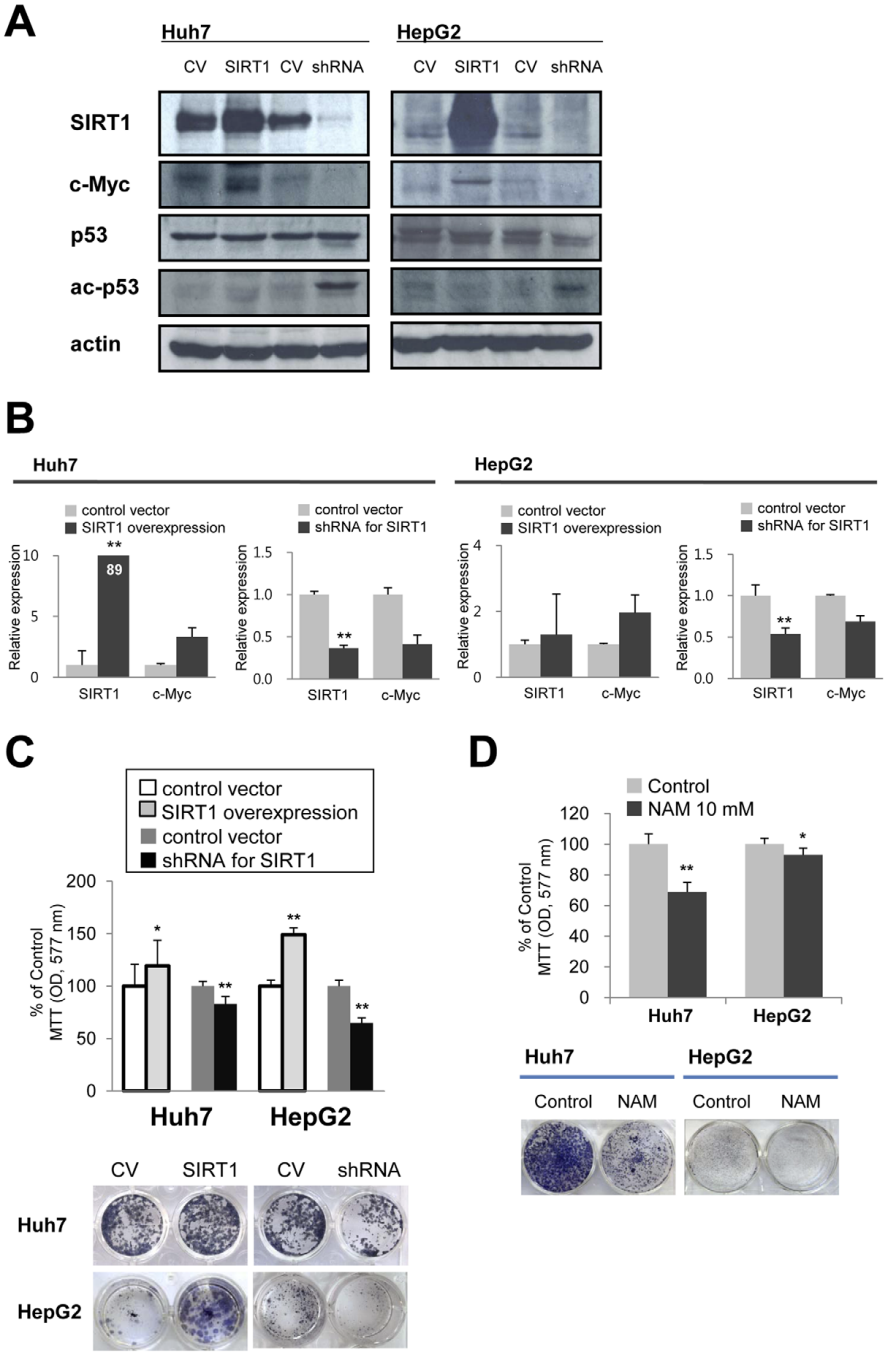
The effect of SIRT1 on the proliferation of human hepatocellular carcinoma cell lines. A) Over-expression and knock-down of SIRT1 was induced in the Huh7 and HepG2 human hepatocellular carcinoma (HCC) cell lines using constructs as described above. Over-expression of SIRT1 increased the expression of c-Myc protein. Knock-down of SIRT1 resulted in relatively decreased expression of c-Myc protein and a detectable increase in the acetylation of the p53 protein. B) qPCR for SIRT1 and c-Myc after induction of over-expression and knock-down of SIRT1. C) SIRT1 over-expression results in increased proliferation of HCC cells. The proliferation of SIRT1 knock-down HCC cells is significantly decreased compared with control cells as determined by both MTT assay and colony forming assay. D) Treatment of HCC cells with NAM results in significant decreased proliferation of both Huh7 and HepG2 cells by both MTT and colony forming assay. MTT assay was performed after seeding of 2×10^3^ cells for two days. A colony-forming assay was performed by culture 2×10^3^ cells for 7 days in 24-well culture plates. A single asterik indicates *P*<0.05, two asteriks indicate *P*<0.001.

Since it has been previously reported that the biologic effects of SIRT1 may vary according to species and tumor types, we next sought to determine the effect of SIRT1 manipulation in two well characterized human HCC cell lines. In general the effect of overexpression or knock down of SIRT1 was in line with the results observed in the mouse derived Tet-O-MYC liver tumor cells. The overexpression of SIRT1 induced increased expression of c-Myc whereas the shRNA mediated knock down of SIRT1 resulted in decreased c-Myc expression (Figure 3 A and B). Moreover, knock-down of SIRT1 resulted in a detectable increase in acetylated p53 (Figure 3 A). Specifically, with SIRT1 overexpression, cells were more proliferative than control cells and the proliferation of cells subjected to SIRT1 knock-down decreased compared with control cells (Figure 3 C). Furthermore, the chemical inhibitor NAM decreased the proliferation and colony forming capacity of both human HCC cell lines (Figure 3 D).

### SIRT1 Expression in c-Myc and DDC Induced Liver Tumors and the Effect of SIRT1 Inhibition on Liver Tumor Development in Mice

**Figure 4:**
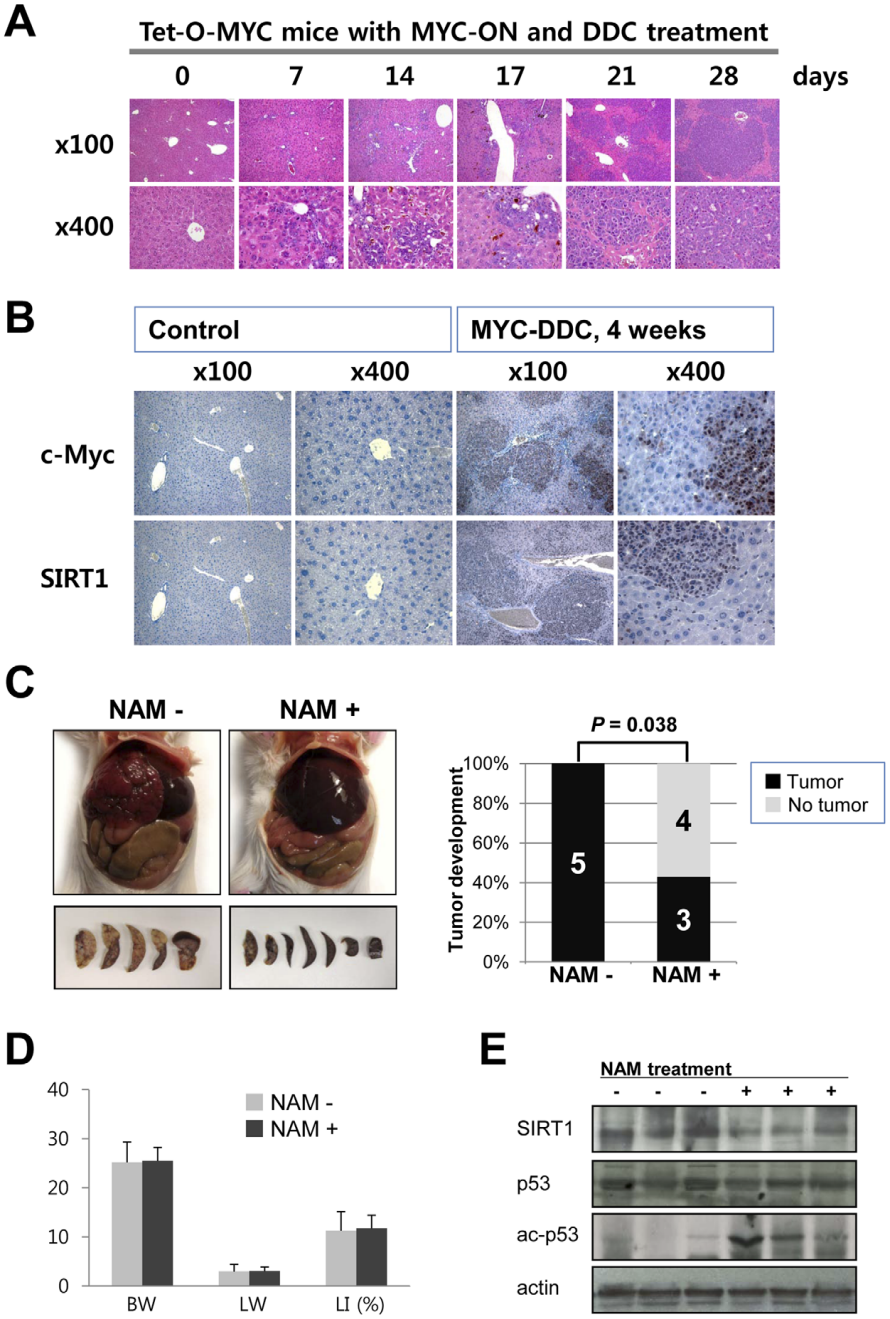
SIRT1 expression in liver tumors and the inhibition of SIRT1 decreases hepatic tumorigenesis in mice. A) Seven days after induction of c-Myc overexpression along with 3,5-diethoxycarbonyl-1,4-dihydrocollidine (DDC) treatment, small atypical cell foci appear and tumor nests gradually increase in size. B) Immunohistochemical staining for c-Myc and SIRT1 shows absent expression of c-Myc and SIRT1 in hepatocytes of control mice without c-Myc activation. In parallel with the development of hepatic tumors in MYC-DDC mice, the expression of c-Myc and SIRT1 increase in tumor cells. Tumor cells demonstrate strong expression of c-Myc and SIRT1 in comparison to adjacent non-transformed hepatocytes. C) All five MYC-DDC mice without nicotinamide (NAM) treatment developed grossly visible multinodular hepatic tumors. Conversely, only three of seven MYC-DDC mice treated with NAM (10 mg/mL) developed hepatic tumor (*P* = 0.038). D) Body weight (BW), liver weight (LW), and liver index LI, (liver weight/body weight) x100] in DDC treated c-Myc transgenic mice (MYC-DDC mice) with or without administration of NAM. There is no difference of BW, LW, and LI between control and NAM administrated MYC-DDC mice. E) Administration of NAM resulted in a detectable increase in acetylation of p53 protein.

As previously reported [24], the non-oncogenic hepatotoxin 3,5-diethoxycarbonyl-1,4-dihydrocollidine (DDC) along with c-Myc over-expression in a mouse model reproducibly results in uniform liver tumor development within four weeks of initiation. We sought to exploit the predictable kinetics of the MYC/DDC tumor model system to determine the relationship of MYC and SIRT1 *in vivo*. Seven days after induction of c-Myc overexpression in conjunction with DDC treatment, small proliferating atypical cell foci appeared (Figure 4 A). 21 days after induction of MYC-DDC, coalescing tumor cells form grossly identifiable large tumor nests (Figure 4 A). Tumor cell volumes were noted to be small with increased nuclear to cytoplasmic ratios, and with prominent nucleoli. Frequent mitoses were also identifiable (Figure 4 A). Since the expression of SIRT1 strongly correlated with c-Myc expression in the Tet-O-MYC cells, we examined the expression of c-Myc and SIRT1 in MYC-DDC tumors (Figure 4 B). In control liver, c-Myc and SIRT1 was not detectable in normal hepatocytes. However, expression of c-Myc and SIRT1 expression was significantly increased in hepatic tumors in MYC-DDC mouse liver. Specifically, tumor cells demonstrated stronger expression of c-Myc and SIRT1 than adjacent non-transformed hepatocytes (Figure 4 B). Next, we investigated the effect of SIRT1 inhibition on liver tumorigenesis *in vivo*. In the control group, all five MYC-DDC mice without treatment by NAM developed liver tumors at four weeks. Conversely, the incidence of tumor development in the liver of MYC-DDC mice was significantly attenuated by treatment with NAM (*P* = .038) (Figure 4 C). Only three of seven NAM treated MYC-DDC mice developed liver tumors. Two of three had grossly detectable liver tumors and one mouse had only microscopically detectable tumor. Between the control and NAM treated groups, there was no difference in either body or liver weight (Figure 4 D). Histologically, there was no evidence of NAM related toxicity in the liver of NAM treated mice. NAM treatment in MYC-DDC mice resulted in increased acetylation of the tumor suppressor protein p53 (Figure 4 E).

### Association of SIRT1 and c-Myc Expression with Clinicopathologic Characteristics of HCC Patients

**Figure 5:**
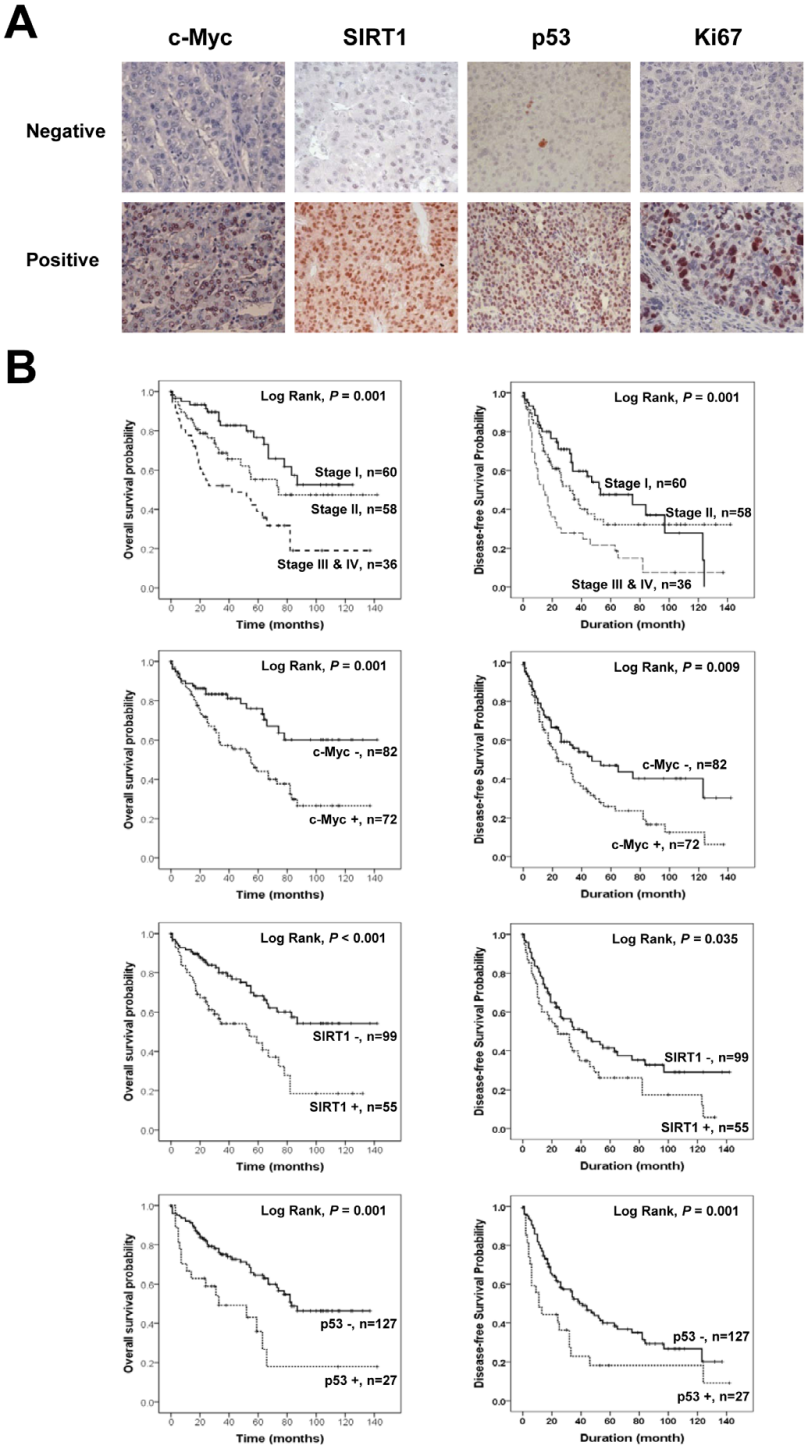
Kaplan-Meier survival analysis in hepatocellular carcinoma. A) Immunohistochemical expression of c-Myc, SIRT1, p53 and Ki67 in human hepatocellular carcinoma tissue (original magnification×400). B) Overall survival (OS) and disease-free survival (DFS) in 154 patients according to the TNM stage, c-Myc expression, SIRT1 expression, and p53 expression.

Next, in order to investigate the potential clinical relevance of these findings, we undertook an examination of c-Myc, SIRT1, p53 and Ki67 expression in a cohort of human HCC tissues. As demonstrated in Figure 5, immunoreactivity for c-Myc, SIRT1, p53 and Ki67 was found primarily in the nuclei of tumor cells. Positive expression of SIRT1, c-Myc, p53 and Ki67 was seen in 36% (55/154), 47% (72/154), 18% (27/154), and 38% (58/154) of patients, respectively (Table 1). The expression of SIRT1 was significantly associated with preoperative serum a-fetoprotein (AFP) levels (*P = *0.032), presence of HBV infection (*P = *0.023), and the expression of c-Myc (*P = *0.014), p53 (*P*<0.001) and Ki67 (*P = *0.011). The correlation coefficient between c-Myc and SIRT1 expression was 0.198 (*P = *0.014). The expression of c-Myc significantly correlated with preoperative serum albumin levels (*P = *0.011) and the TNM stage (*P = *0.02). The expression of p53 also significantly associated with serum AFP level (*P = *0.036), HBV infection (*P = *0.002), and serum bilirubin level (*P = *0.03). The expression of Ki67 significantly associated with serum AFP level (*P = *0.005), HBV infection (*P = *0.03), and high histologic grade (*P = *0.04). Additional variables detailed in Table 1 displayed no statistically significant association with the expression of SIRT1, c-Myc, p53, or Ki67 in HCCs.

**Table 1:** Clinicopathologic variables and the expression status of SIRT1, c-Myc, p53, and Ki67.

			SIRT1		c-Myc		p53		Ki67	
Characteristics		Total	Positive	*P*	Positive	*P*	Positive	*P*	Positive	*P*
Sex	Male	132	47 (36%)	0.945	64 (48%)	0.291	20 (15%)	0.057	51 (39%)	0.541
	Female	22	8 (36%)		8 (36%)		7 (32%)		7 (32%)	
Age, y	<55	66	22 (33%)	0.593	31 (47%)	0.963	13 (20%)	0.541	24 (36%)	0.773
	≥55	88	33 (38%)		41 (47%)		14 (16%)		34 (39%)	
AFP, ng/mL	<100	101	30 (30%)	0.032	45 (45%)	0.45	13 (13%)	0.036	30 (30%)	0.005
	≥100	53	25 (47%)		27 (51%)		14 (26%)		28 (53%)	
HBV	Negative	42	9 (21%)	0.023	20 (48%)	0.895	1 (2%)	0.002	10 (24%)	0.03
	Positive	112	46 (41%)		52 (46%)		26 (23%)		48 (43%)	
HCV	Negative	144	52 (36%)	0.697	70 (49%)	0.08	27 (19%)	0.132	54 (38%)	0.875
	Positive	10	3 (30%)		2 (20%)		0 (0%)		4 (40%)	
Liver cirrhosis	Absence	81	31 (38%)	0.485	42 (52%)	0.182	15 (19%)	0.735	33 (41%)	0.406
	Presence	73	24 (33%)		30 (41%)		12 (16%)		25 (34%)	
Bilirubin, mg/dl	<0.7	69	24 (35%)	0.828	33 (48%)	0.81	7 (10%)	0.03	27 (39%)	0.735
	≥0.7	85	31 (36%)		39 (46%)		20 (24%)		31 (36%)	
Albumin, ng/dl	<3.5	17	5 (29%)	0.565	3 (18%)	0.011	2 (12%)	0.507	8 (47%)	0.397
	≥3.5	137	50 (36%)		69 (50%)		25 (18%)		50 (36%)	
TNM stage	I	60	21 (35%)	0.893	26 (43%)	0.02	8 (13%)	0.506	19 (32%)	0.329
	II	58	22 (38%)		22 (38%)		11 (19%)		26 (45%)	
	III & IV	36	12 (33%)		24 (67%)		8 (22%)		13 (36%)	
Histologic grade	Low	93	31 (33%)	0.446	47 (51%)	0.245	12 (13%)	0.062	29 (31%)	0.04
	High	61	24 (39%)		25 (41%)		15 (25%)		29 (48%)	
Ki67	Negative	96	27 (28%)	0.011	47 (49%)	0.48	13 (14%)	0.094		
	Positive	58	28 (48%)		25 (43%)		14 (24%)			
p53	Negative	127	37 (29%)	<0.001	59 (46%)	0.873				
	Positive	27	18 (67%)		13 (48%)					
c-Myc	Negative	82	22 (27%)	0.014						
	Positive	72	33 (46%)							

Abbreviations: AFP, á-fetoprotein.

### Expression of SIRT1 and c-Myc Predicts Shorter Survival of HCC Patients

We next sought to determine the relationship if any of oncoprotein SIRT1, c-Myc, p53, and Ki67 to OS and DFS as shown in Table 2. Advanced tumor stage (*P = *0.001), serum AFP level (*P = *0.008), serum albumin level (*P = *0.029), as well as the expression of SIRT1 (*P*<0.001), c-Myc (*P = *0.001), and p53 (*P = *0.001) were all significantly associated with shorter OS according to univariate analysis Advanced tumor stage (*P = *0.001), serum AFP level (*P = *0.001), serum albumin level (*P = *0.017), high histologic grade (*P* = 0.014) and expression of SIRT1 (*P*<0.039), c-Myc (*P = *0.011), and p53 (*P = *0.006) were associated with significantly shorter DFS according to univariate analysis (Figure 5 B).

**Table 2:** Univariate Cox regression analysis for overall survival and disease-free survival in hepatocellular carcinoma patients.

Characteristics	No.		OS			DFS	
		HR	95% CI	*P*	HR	95% CI	*P*
Age ≥55 y (*vs* <55 y)	88/154	1.311	0.787–2.183	0.298	1.284	0.853–1.931	0.231
Sex female (*vs* male)	22/154	0.861	0.410–1.807	0.692	0.97	0.540–1.743	0.92
HBV positive (*vs* negative)	112/154	1.111	0.631–1.957	0.716	1.221	0.764–1.951	0.404
HCV positive (*vs* negative)	3/154	1.451	0.582–3.619	0.425	1.838	0.888–3.803	0.101
AFP≥100 ng/ml (*vs* <100 ng/ml)	53/154	1.952	1.192–3.196	0.008	1.968	1.317–2.943	0.001
Albumin <3.5 ng/dl (*vs* ≥3.5 ng/dl)	17/154	2.129	1.081–4.190	0.029	2.05	1.139–3.690	0.017
Bilirubin ≥0.7 mg/dl (*vs* <0.7 mg/dl)	85/154	1.274	0.775–2.095	0.339	1.078	0.722–1.611	0.712
Stage I	60/154			0.001			0.001
II	58/154	1.657	0.879–3.123	0.118	1.278	0.784–2.082	0.325
III & IV	36/154	3.134	1.689–5.816	<0.001	2.49	1.516–4.090	<0.001
Cirrhosis presence (*vs* absence)	73/154	1.013	0.620–1.655	0.958	0.928	0.622–1.384	0.714
Histologic grade high (*vs* low)	61/154	1.598	0.970–2.631	0.066	1.654	1.105–2.476	0.014
SIRT1 positive (*vs* negative)	55/154	2.518	1.536–4.128	<0.001	1.532	1.023–2.294	0.039
c-Myc positive (*vs* negative)	72/154	2.354	1.397–3.969	0.001	1.692	1.130–2.533	0.011
p53 positive (*vs* negative)	27/154	2.501	1.427–4.384	0.001	1.97	1.219–3.184	0.006
Ki67 positive (*vs* negative)	58/154	0.976	0.588–1.619	0.924	1.21	0.807–1.815	0.355

Abbreviations: OS, overall survival; DFS, disease-free survival; AFP, á-fetoprotein; HR, hazard ratio.

Next, multivariate analysis was performed using all patients with complete information for all variables (Table 3). Variables significantly associated with OS and DFS upon univariate Cox regression analysis were included in the multivariate analysis. The subjects with SIRT1 expression had a 2.144-fold (95% CI, 1.265–3.635) greater risk of death (*P = *0.005) and the patients with c-Myc expression had a 2.902-fold (95% CI, 1.598–5.269) greater risk of death (*P*<0.001). The expression of c-Myc was also predictive of reduced DFS with a *P* value of 0.006 (hazard ratio, 1.851; 95% CI, 1.19–2.879). SIRT1 expression was not associated with DFS according to multivariate analysis (*P* = 0.474). Tumor stage, serum albumin level, and p53 expression were also independent prognostic factors significantly associated with OS and DFS according to multivariate analysis.

**Table 3:** Multivariate Cox regression analysis for overall survival and disease-free survival in hepatocellular carcinoma patients.

Characteristics		OS			DFS	
	HR	95% CI	*P*	HR	95% CI	*P*
Stage I			0.001			0.002
II	2.723	1.374–5.396	0.004	1.668	0.977–2.849	0.061
III & IV	3.271	1.746–6.131	<0.001	2.537	1.506–4.274	<0.001
Albumin <3.5 ng/dl (*vs* ≥3.5 ng/dl)	4.753	2.173–10.394	<0.001	3.71	1.918–7.179	<0.001
AFP≥100 ng/ml (*vs* <100 ng/ml)	1.505	0.889–2.549	0.128	1.732	1.143–2.623	0.01
c-Myc positive (*vs* negative)	2.902	1.598–5.269	<0.001	1.851	1.190–2.879	0.006
SIRT1 positive (*vs* negative)	2.144	1.265–3.635	0.005	1.186	0.743–1.892	0.474
p53 positive (*vs* negative)	2.081	1.149–3.768	0.016	2.24	1.363–3.682	0.001

Abbreviations: OS, overall survival; DFS, disease-free survival; AFP, á-fetoprotein; HR, hazard ratio. Variables significantly associated with OS and DFS upon univariate Cox regression analysis were included in the multivariate analysis. Variables considered for OS analysis were TNM stage, AFP level, albumin level, SIRT1 expression, c-Myc expression, and p53 expression. Variables considered for DFS analysis were TNM stage, AFP level, albumin level, histologic grade, SIRT1 expression, c-Myc expression, and p53 expression.

## Discussion

In the present study the role of SIRT1 in liver cancer was investigated. Studies included an examination of the biologic effect of SIRT1 on cell proliferation and survival as well as an analysis of SIRT1 as a possible prognostic factor in human HCC. We utilized a conditional transgenic mouse and cell line for the controlled expression of c-Myc to investigate the role of SIRT1 expression in c-Myc driven liver tumorigenesis. We also investigated the possible clinical significance of our findings in a cohort of human HCC tumor samples. Of significance, we report that: (1) SIRT1 expression is tightly correlated with oncogene (c-Myc) expression and promotes the proliferation of liver cancer cells; (2) genetic or physio-chemical inhibition of SIRT1 with shRNA to SIRT1 or NAM respectively, inhibits the proliferation of liver cancer cells; (3) SIRT1 inhibition *in vivo* with NAM significantly reduces tumorigenesis in a mouse tumor model; (4) SIRT1 expression is significantly associated with the expression of c-Myc, p53, and Ki67; and (4) the expression of SIRT1 and c-Myc is associated with a significantly shortened survival in HCC patients. Taken together, our findings suggest that SIRT1 cooperates with c-Myc for the progression of HCCs and influences the prognosis of HCC patients.

The role of SIRT1 in tumorigenesis is controversial especially in the association between SIRT1 and oncogenic c-Myc. Recently, positive correlation between c-Myc and SIRT1 in tumorigenesis has been reported [6], [7]. In colorectal cancer, c-Myc increased both SIRT1 protein level and deacetylase activity of SIRT1 [7]. In addition, SIRT1 also increased transcriptional activity of c-Myc and its stabilization in fibroblasts [6], [7]. SIRT1 mediated deacetylation of c-Myc increased the half-life of c-Myc and promoted c-Myc/Max association that resulted in suppression of cellular senescence and inhibition of apoptosis [6], [7]. In contrast, one early report has shown that SIRT1 de-stabilized c-Myc by deacetylation despite increasing c-Myc transcription and therefore suggesting SIRT1 as a negative regulator of Myc directed tumorigenesis [8]. In the present study, we demonstrate that the expression of SIRT1 and c-Myc are positively correlated and both promote the proliferation of tumor cells. Induced c-Myc expression in Tet-O-MYC cells results in a corresponding increased expression of SIRT1 and overexpression of SIRT1 also produced increased c-Myc expression. Conversely, oncogenic c-Myc silencing resulted in a significant and tightly corresponding decrease in expression of SIRT1. Intriguingly, oncogenic c-MYC re-activation resulted in the prompt reversal of SIRT1 expression. The biologic effect of SIRT1 modulation in human HCC cell lines was identical to that observed in the mouse Tet-O-MYC cells. Moreover, SIRT1 expression in human HCC significantly correlated with the expression of c-Myc and Ki67. Together these findings support a consistent role for SIRT1 and MYC expression in both mouse and human hepatocellular proliferation and survival. When considering the role of c-Myc as a potent oncogene, our results suggest that SIRT1 can be regarded as an important target of c-Myc in liver tumorigenesis.

The data presented herein also demonstrate that SIRT1 regulates the acetylation status of p53 in tumor cells and the expression of SIRT1 significantly correlates with p53 expression in HCC tissue samples. SIRT1 has previously been suggested as a potential tumorigenic factor through its deacetylation of p53 leading to its inactivation [5] and inhibition of apoptosis [25]. In MYC-ON cells the pro-proliferative effects of SIRT1 over-expression likely relate to a mechanism of SIRT1-mediated inhibition of p53 in addition to the effects on oncogenic c-Myc. As previously shown, c-Myc expression potently induces both cellular proliferation and apoptosis [26], [27]. In the Tet-O-MYC system, we observed cleaved caspase-3 expression is co-temporally related to the MYC expression status (Figure S1 A and B). Confirming these findings, MYC driven tumors *in vivo* demonstrate high numbers of both mitotic and apoptotic cells (Figure S1 C). Conversely, the expression of cleaved caspase-3 decreased with SIRT1 over-expression (Figure S1 D). Therefore, SIRT1 over-expression may also promote cell survival through the inhibition of p53-mediated apoptosis. Our results also demonstrate that knock down of SIRT1 in liver cancer cells induces a detectable increase in acetylated p53 along with an overall decrease in the proliferative activity of cells. Moreover, increased acetylation of p53 in NAM treated MYC-DDC mice *in vivo* were accompanied by decreased hepatocellular tumorigenesis. Accordingly, our findings suggest that SIRT1 may contribute to liver tumorigenesis at least in part by controlling cellular proliferation and survival through the modulation of p53 activity. These observations also support and may provide a plausible contributing mechanism for our observation that patients with SIRT1 expressing tumors possess an unfavorable prognosis. However, the role of SIRT1 in liver tumorigenesis remains incompletely understood given the numerous biologic roles that have been described for the sirtuins. Moreover, the relationship between SIRT1 and p53 remains complex since the mutational status of p53 may alter the biologic influence of SIRT1 on p53 activity. As we have shown in Figure 3, the knockdown of SIRT1 in Huh7 cells showed more effect on suppression of c-Myc mRNA than HepG2 cells. However, the effect of SIRT1 knockdown in Huh7 cell has a smaller effect on cell proliferation compared to the effect in HepG2 cells. This discrepancy may relate to the mutational status of p53 in Huh7 and HepG2 cells. Huh7 cells [28] are mutant for p53 and HepG2 cells [29] are wild type for p53. In Huh7 cells, suppression of c-Myc by SIRT1 knockdown may induce inhibition of cell proliferation. However, the influence of SIRT1 knockdown to mutated p53 of Huh7 cell is unpredictable despite our demonstration that of SIRT1 knockdown increases p53 acetylation in Huh7 cells. Therefore, we propose that the combined effects of SIRT1 knockdown on c-Myc and mutant p53 likely results in a diminished effect on the proliferation of Huh7 cells. In contrast, the inhibitory effect of SIRT1 knockdown on HepG2 cells may relate primarily to the inhibition of wild type p53 by deacetylation despite limited measurable effect on c-Myc expression. Additional studies beyond the scope of this work are needed to clarify the exact role of SIRT1 relative to p53 mutational status.

The role of SIRT1 in tumorigenesis is controversial. Our study has shown that SIRT1 acts primarily as a tumor promoting factor in hepatocellular carcinogenesis. Together our data demonstrate at least three possible mechanisms to account for this tumor promoting activity. Our clinical sample analysis suggests a strong positive correlation between SIRT1 and oncogenic MYC. Moreover, we have provided *in vitro* evidence that SIRT1 promotes oncogenic c-MYC expression simultaneous with a suppressive effect of oncogene induced apoptosis through the p53 axis. However, an additional potential mechanistic explanation for SIRT1 tumor promoting activity may involve the activation of oncogenes in addition to c-Myc. In contrast to previous reports that SIRT1 negatively regulates oncoprotein â-catenin in colon cancer [21] and survivin in breast cancer [30], in our studies SIRT1 overexpression induced increased â-catenin mRNA and protein expression, increased â-catenin TCF/LEF transcription co-activation and increased expression of â-catenin target genes cyclin D1 and survivin in liver tumor cells (Figure S2). Therefore, we propose that the combined effects of increased SIRT1 signaling on various oncogenes such as c-Myc and â-catenin, is multifactorial and at least in part is protection against apoptosis involving survivin and p53 during hepatic tumorigenesis.

In order to begin to investigate whether SIRT1 activity could be manipulated to an observable effect *in vivo*, we undertook a series of experiments to inhibit SIRT1 activity using its physiologic inhibitor NAM. Accordingly, in a tumor prevention approach, we found that the administration of NAM in DDC treated c-Myc transgenic mice significantly decreased the incidence of liver tumorigenesis. In addition to the role of NAM as a SIRT1 inhibitor, NAM may also function as an antioxidant. Therefore, in order to account for the possibility that NAM was exacting a predominant antioxidant effect on liver tumorigenesis as had been previously described, we tested the effect of the established antioxidant *N*-acetyl-L-cysteine (NAC) on liver tumorigenesis. We administrated NAC (10 mg/mL) in the drinking water to four MYC-DDC mice. We observed that NAC did not noticeably influence liver tumorigenesis as all four NAC treated mice developed liver tumors (Figure S3). Consequently, we conclude the antitumor effects of NAM are likely independent of an anti-oxidant function and therefore more likely correlate with the inhibitory effects of SIRT1.

In the course of our studies, we were also interested in extending our observations to an established cohort of archived human HCC tumor samples. SIRT1 expression previously reported between 31% and 56% of HCC [14], [23], [31], and in this study 36% of HCC showed SIRT1 expression. In line with the positive correlation between c-Myc and SIRT1 expression in Tet-O-MYC liver cancer cells, we detected a significant immunohistochemical relationship between SIRT1 and c-Myc in human HCCs. Moreover, the expression of SIRT1 and c-Myc predicted shorter OS and DFS of HCC patients. Together these findings suggest that SIRT1 appears to possess an important role in liver tumorigenesis. In addition, c-Myc expression revealed a significant relationship with established unfavorable clinicopathologic features of HCC patients including low serum albumin and advanced clinical stage. Moreover, when we analyzed the prognostic impact of c-Myc and SIRT1 for HCC patients with the combination of these two molecules, patients expressing both c-Myc and SIRT1 displayed shorter OS and DFS compared with the patients that did not express both c-Myc and SIRT1 (mean overall survival time; 48.45±7.28, 115.08±7.66 months, respectively) (Figure S4). There was also a population of tumors expressing either c-Myc or SIRT1 (c-Myc-/SIRT1+ or c-Myc+/SIRT1−) and these subjects showed a poorer prognosis than the c-Myc-/SIRT1− group. As demonstrated in Figure S4, the survival curve of c-Myc-/SIRT1+ or c-Myc+/SIRT1− groups fell between the c-Myc+/SIRT1+ and c-Myc-/SIRT1− groups. Ten-year survival rate of c-Myc-/SIRT1−, c-Myc-/SIRT1+, c-Myc+/SIRT1−, and c-Myc+/SIRT1+ groups were 70%, 25%, 31%, and 14%, respectively. Therefore, these data suggest there is a correlation between c-Myc and SIRT1 in hepatic tumorigenesis as well as an independent role of c-Myc and SIRT1 in hepatic tumorigenesis. Similar to our results, it has previously been reported that c-Myc expression correlates with the progression and poor prognosis of HCC [32], [33]. However, still it is not clear whether SIRT1 is real prognostic indicator of HCC or not. One recent report suggested SIRT1 as a possible prognostic indicator of HCC [14] but other report could not find prognostic significance of SIRT1 despite higher expression of SIRT1 in cancer tissue compared to adjacent normal tissue [31].

From a clinicopathologic perspective, in this study we also observed that SIRT1 expression significantly correlated with preoperative AFP levels. The serum level of AFP in SIRT1 positive patients (mean ± standard error, 4336±1890) was markedly higher than in SIRT1 negative patients (mean ± standard error, 987±405) (*t*-test, 2-tailed, *P = *0.027). This positive correlation between SIRT1 and serum AFP levels further supports a likely role for SIRT1 in HCC development and progression. Interestingly, SIRT1 expression also significantly correlated with HBV infection. When considering the previous reports that HBV X protein is involved in liver tumorigenesis by inducing the expression of histone deacetylase 1 [34], it raises the intriguing possibility that SIRT1 is also contributing to liver tumorigenesis through its activity as a histone deacetylase induced in HBV infected patients. Further study beyond the scope of this work is warranted to further define the precise tumor promoting mechanism provided by SIRT1.

In summation, our results suggest that SIRT1 cooperates with c-Myc in liver tumorigenesis and may be an important molecular target for modulating liver cancer. As previously suggested by others [35]–[38], in this study we also find that inhibition of SIRT1 may be an effective strategy for the prevention of tumor development in high-risk patients or as a therapeutic strategy in established tumors. In conclusion, our findings indicate that SIRT1 and c-Myc may be involved in the progression of HCCs and both of these molecules may be useful as clinical indicators of overall HCC prognosis.

## Materials and Methods

### Ethics

All animal experiments were performed in accordance with a protocol approved by the Stanford University’s Administrative Panels on Laboratory Animal Care. This study was conducted with institutional review board approval. Informed consent was provided according to the Declaration of Helsinki.

### Transgenic Mice and Cell Lines

In this study we utilized bitransgenic mice that conditionally express the c-MYC proto-oncogene in hepatocytes [3], [39]. c-MYC expression is activated by removing doxycycline treatment (100 µg/ml) from the drinking water of mice. To investigate the role of SIRT1 under the influence of c-Myc *in vitro*, we utilized a primary culture tumor cell line generated from the liver tumors of Tet-O-MYC mice [3], [39]. The human HCC cell lines Huh7 and HepG2 were also utilized. All cell lines were cultured in Dulbecco’s minimal essential medium. Nicotinamide (NAM; Sigma Aldrich, St. Louis, MO) was dissolved in distilled water.

### Genotyping with Polymerase Chain Reaction

DNA was isolated from the tail of the mice or Tet-O-MYC cells using the Qiaprep DNeasy kit (Qiagen, Valencia, CA) in accordance with the manufacturer’s directions. The LAP-tTa segment was detected using the following primers: LAP-tTa forward 5¢-GCTGCTTAATGAGGTCGG-3¢ and LAP-tTa reverse 5¢-CTCTGCACCTTGGTGATC-3¢. The TetO-Myc construct was detected with the following primers: c-Myc forward 5¢-CCTACCCTCTCAACGACAGC-3¢ and c-Myc reverse 5¢-CTCTGACCTTTTGCCAGGAG-3¢. DNA was amplified using the following polymerase chain reaction (PCR) protocol: 94°C denaturation for 2 minutes followed by 35 cycles of 94°C for 15 seconds, 59°C annealing for 30 seconds, and 72°C for 30 seconds, followed by a 5 minute extension at 72°C. PCR products were resolved on a 1.5% gel.

### Tumorigenecity Assays

To accelerate liver tumorigenesis in Tet-O-MYC mice, we fed a diet containing 0.1% DDC (Sigma Aldrich, St. Louis, Missouri, United States) in Tet-O-MYC mice as previously described (MYC-DDC mice).^24^ Five MYC-DDC mice were sacrificed at 7, 14, 17, 21, and 28 days after being fed a DDC enriched diet with c-Myc activation by removing doxycycline from the drinking water. An additional twelve MYC-DDC mice were used for a determination of the effect of NAM on liver tumorigenesis in MYC-DDC mice. Seven MYC-DDC mice were administrated NAM by adding 10 mg/mL NAM into the drinking water and five mice did not receive NAM. Thereafter, mice were sacrificed at four weeks and evaluated for tumor development. Liver tissues were snap frozen in liquid nitrogen or were prepared for histology by fixing in 10% buffered formalin and embedded in paraffin. Paraffin sections were stained with hematoxylin and eosin.

### Transfection of SIRT1 Plasmid DNA and SIRT1 Short Hairpin RNA Plasmid

Human SIRT1 plasmid DNA (pDNA) (Addgene, plasmid 1769, Cambridge, MA) was cloned into pCMV-tag2B (Stratagene, La Jolla, CA). The same plasmid without the SIRT1 DNA insert was used as a control vector (CV). Plasmid for SIRT1 short hairpin RNA (shRNA) and CV was kindly provided by A. Brunet (Stanford University, Stanford, CA). All plasmids were amplified in DH5á *Escherichia coli* competent cells (Invitrogen, Carlsbad, CA) and were purified using an endo-free plasmid mega-prep kit (Qiagen, Valencia, CA). Transient transfection of Tet-O-MYC, Huh7 and HepG2 cells with human SIRT1 pDNA and shRNA was performed using Lipofectamin 2000 (Invitrogen, Carlsbad, CA). 44 hours after transfection, the cells were harvested and used for further experiments.

### Cell Proliferation Assay

The number of live cells was counted by using trypan blue exclusion. For the assessment of cell proliferation and survival/viability, a commercially available 3-(4,5-dimethylthiazol-2-yl)-2,5-diphenyltetrazolium bromide (MTT) cell proliferation assay (Roche Applied Science, Indianapolis, IN) and colony-forming assay were utilized. The MTT assay was performed after seeding of 2×10^3^ cells for two to three days. Absorbance was measured at 577 nm. The colony forming assay was performed by cell culture in 24 or 6 well culture plates.

### Quantitative Reverse-transcription Polymerase Chain Reaction

RNA isolation was performed with the RNeasy Mini Kit (Qiagen Sciences, Valencia, CA). After DNase treatment, reverse transcription of 1.5 µg RNA was performed with Taqman Reverse Transcription Reagents (Applied Biosystems, Foster City, CA). Quantitative real-time polymerase chain reaction (qRT-PCR) was carried out using the Applied Biosystems Prism 7900HT Sequence Detection System and Sybr Green or Taqman PCR Master Mix (Applied Biosystems, Foster City, CA). All experiments were performed in triplicate and the results were normalized to the expression of the glyceraldehyde-3-phosphate dehydrogenase (GAPDH) reference housekeeping gene. Primer sequences for PCR were: c-Myc forward 5¢-CTGCGACGAGGAGGAGGACT-3¢, reverse 5¢- GGCAGCAGCTCGAATTTCTT-3¢; SIRT1 forward 5′-TGCTGGCCTAATAGAGTGGCA-3′, reverse 5′-CTCAGCGCCATGGAAAATGT-3′; â-catenin forward 5′-GTCAGCTCGTGTCCTGTGAA-3′, reverse 5′-GATCTGCATGCCCTCATCTA-3′; cyclin D1 forward 5′-TGGAGCCCCTGAAGAAGAG-3′, reverse 5′-AAGTGCGTTGTGCGGTAGC -3′; survivin forward 5′-GGACCACCGCATCTCTACAT -3′, reverse 5′-GCACTTTCTTCGCAGTTTCC-3′; GAPDH forward 5¢-GACGGCCGCATCTTCTTGT-3¢, reverse 5¢-CACACCGACCTTCACCATTTT-3¢.

### Luciferase Reporter Assay

Dual-Light Reporter Gene Assay (Applied Biosystems, Foster City, CA) was performed using the pMegaTOPFLASH, pMegaFOPFLASH, 5×-HRE, or LacZ plasmids as previously described [40]–[42].

### Western Blot Analysis

Total proteins lysed with RIPA buffer were separated by SDS-PAGE and transfrerred to a PVDF membrane. The blot was probed with primary antibodies for c-Myc (Santa Cruz, clone C-19, CA), SIRT1 (Santa Cruz, clone H-300, CA), p53 (Santa Cruz, clone FL-393, CA), acetyl-p53 (Lys382) (Cell Signaling Technology, Beverly, MA), acetyl-p53 (Lys379) (Cell Signaling Technology, Beverly, MA), p21 (Santa Cruz, clone M-19, CA), and β-actin (Abcam, Cambridge, MA).

### Hepatocellular Carcinoma Patients and Tissue Samples

154 cases of HCC patients who underwent radical resection in Chonbuk National University Hospital between January 1998 and August 2009, and for whom initial diagnostic paraffin-embedded tissue blocks were available were included in the present study. All of the cases were reviewed and reclassified according to the criteria of the World Health Organization Classification [43]. Pathologic staging was reviewed based on the staging system of the American Joint Committee on Cancer [44]. The patients grouped according to sex, age (≥55 *versus* <55), clinical stage (I *versus* II *versus* III and IV), HBV infection, HCV infection, cirrhosis (absence *versus* presence), AFP level (≥100 ng/ml *versus* <100 ng/ml) [43], albumin level (<3.5 ng/dl *versus* ≥3.5 ng/dl; normal range, 3.5–5.3 ng/dl), bilirubin level (≥0.7 mg/dl *versus* <0.7 mg/dl), histologic grade (low *versus* high), and immunohistochemical expression of c-Myc, SIRT1, p53, and Ki67. The cut-off value of bilirubin level was determined by receiver operating characteristic curve analysis at the highest positive likelihood ratio point.

### Immunofluorescence and Immunohistochemical Staining

Immunofluorescence staining was performed in cultured cells after fixation with cold methanol, and then incubated with the primary antibody for 1 hour. FITC-conjugated secondary antibody was used. Immunocytochemistry was performed in cultured cells after fixation with cold methanol. Immunohistochemistry was performed in paraffin-embedded blocks. For tissue microarray (TMA) of human HCC, two 3.0 mm sized cores per case were arrayed. The tissue sections were treated with a microwave antigen retrieval procedure in sodium citrate buffer. The following markers were used: c-Myc (Santa Cruz, clone 9E10, CA), SIRT1 (Santa Cruz, clone H-300, CA), Ki67 (DAKO, clone MIB1, Denmark), and p53 (Novocastra, clone DO-7, Newcastle, UK).

### Immunohistochemical Scoring for Tissue Microarray

Immunohistochemical analysis of TMA blocks was performed by three pathologists by consensus without knowledge of the clinicopathological information. Immunostaining for c-Myc and SIRT1 was considered positive if 30% or more of the tumor cells stained with an antibody [15]–[17], [45]. For p53 and Ki67 immunostaining, staining of more than 10% of the cells was regarded as positive.

### Statistical Analysis

All experiments were performed three times and representative data are presented. Student’s *t* test and Pearson’s chi-square test were used for comparision between groups. In the survival analysis, the end point of interest was overall survival (OS) and disease-free survival (DFS). The follow-up end point was the date of the last contact or the date of death through November 2010. OS was calculated as the time from diagnosis to the date of death or last contact. Patients who were alive at last contact were treated as censored for OS analysis. DFS was calculated from the time of diagnosis to the date of first recurrence, latent metastasis, death, or last contact. Patients who were alive at last contact and who had not experienced tumor recurrence or latent metastasis were treated as censored for DFS analysis. Univariate and multivariate Cox proportional hazards regression analyses were performed to estimate the impact of clinicopathologic factors and expression of each marker on OS and DFS. Kaplan-Meier survival curves were constructed to further illustrate the impact of OS and DFS when indicated. SPSS software (version 15.0) was used throughout. *P* values less than 0.05 were considered statistically significant.

## Supporting Information

Figure S1 c-Myc expression and increased apoptosis; SIRT1 expression inhibits apoptosis. A) The expression of cleaved caspase-3 directly correlates with c-MYC in a time dependent manner. When oncogenic c-Myc expression is restored over five days (Re-ON), increased cleaved caspase-3 expression returns. B) MYC-ON cells with increased cleaved caspase-3 by immunocytochemistry. C) The tumor of the MYC-DDC mice shows high numbers of mitosis (empty arrows) and apoptosis (arrow heads) (original magnification x40). D) Over-expression of SIRT1 is associated with a decrease in cleaved caspase-3 expression.
(TIF)

Figure S2 Over-expression of SIRT1 induces â-catenin expression. A) Over-expression of SIRT1 in Tet-O-MYC cells increases β-catenin/TCF reporter activity. B) Quantitative real time PCR. After transfection of Tet-O-MYC cells with a SIRT1 over-expression plasmid, the expression of mRNA for SIRT1, β-catenin, cyclin D1, and survivin significantly increases compared with control vector. C) Over-expression of SIRT1 was induced in the Huh7 and HepG2 human hepatocellular carcinoma cell lines. Over-expression of SIRT1 increases the expression of β-catenin protein. One asteriks indicate *P*<0.05 and two asteriks indicate *P*<0.001.
(TIF)

Figure S3 The effect of *N*-acetyl-L-cysteine in hepatic tumorigenesis. Administration of *N*-acetyl-L-cysteine (NAC) in DDC treated c-Myc transgenic mice (MYC-DDC mice) does not influence liver tumorigenesis. All four MYC-DDC mice with NAC (10 mg/mL) and all five MYC-DDC mice without NAC treatment developed hepatic tumors (*P* = 1.000).
(TIF)

Figure S4 Kaplan-Meier survival analysis in hepatocellular carcinoma according to the combined expression of c-Myc and SIRT1. Overall survival and disease-free survival in 154 patients according to the combined expression of c-Myc and SIRT1
(TIF)
