# Supplementary figures and images for: SIRT1 and c-Myc Promote Liver Tumor Cell Survival and Predict Poor Survival of Human Hepatocellular Carcinomas
Source: PLoS One. 2012 Sep 14;7(9):e45119. doi: 10.1371/journal.pone.0045119 (PMC3443243; doi:10.1371/journal.pone.0045119)

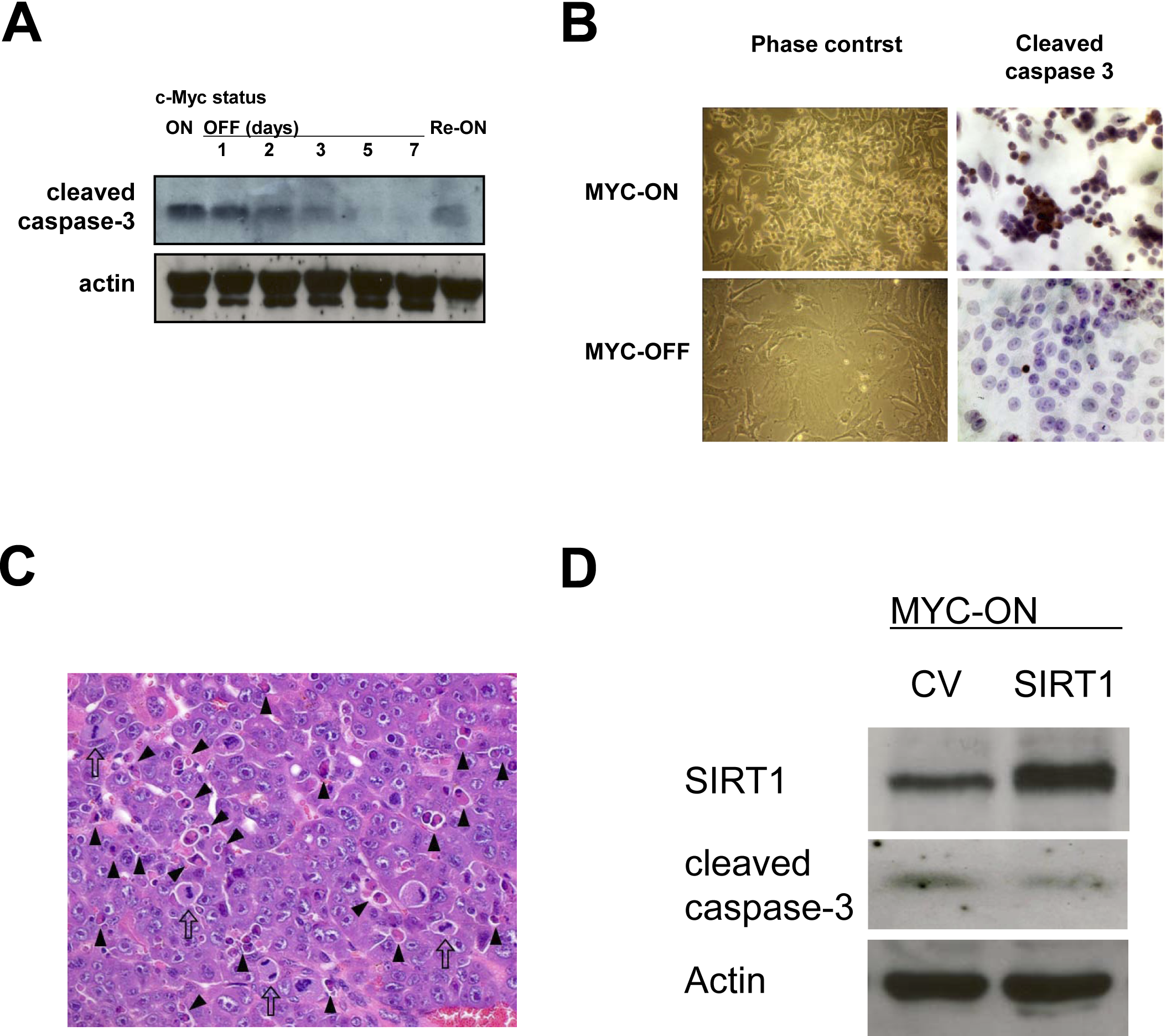

Supplement: Figure S1 — c-Myc expression and increased apoptosis; SIRT1 expression inhibits apoptosis. A) The expression of cleaved caspase-3 directly correlates with c-MYC in a time dependent manner. When oncogenic c-Myc expression is restored over five days (Re-ON), increased cleaved caspase-3 expression returns. B) MYC-ON cells with increased cleaved caspase-3 by immunocytochemistry. C) The tumor of the MYC-DDC mice shows high numbers of mitosis (empty arrows) and apoptosis (arrow heads) (original magnification x40). D) Over-expression of SIRT1 is associated with a decrease in cleaved caspase-3 expression. (TIF) [file pone.0045119.s001.tif]

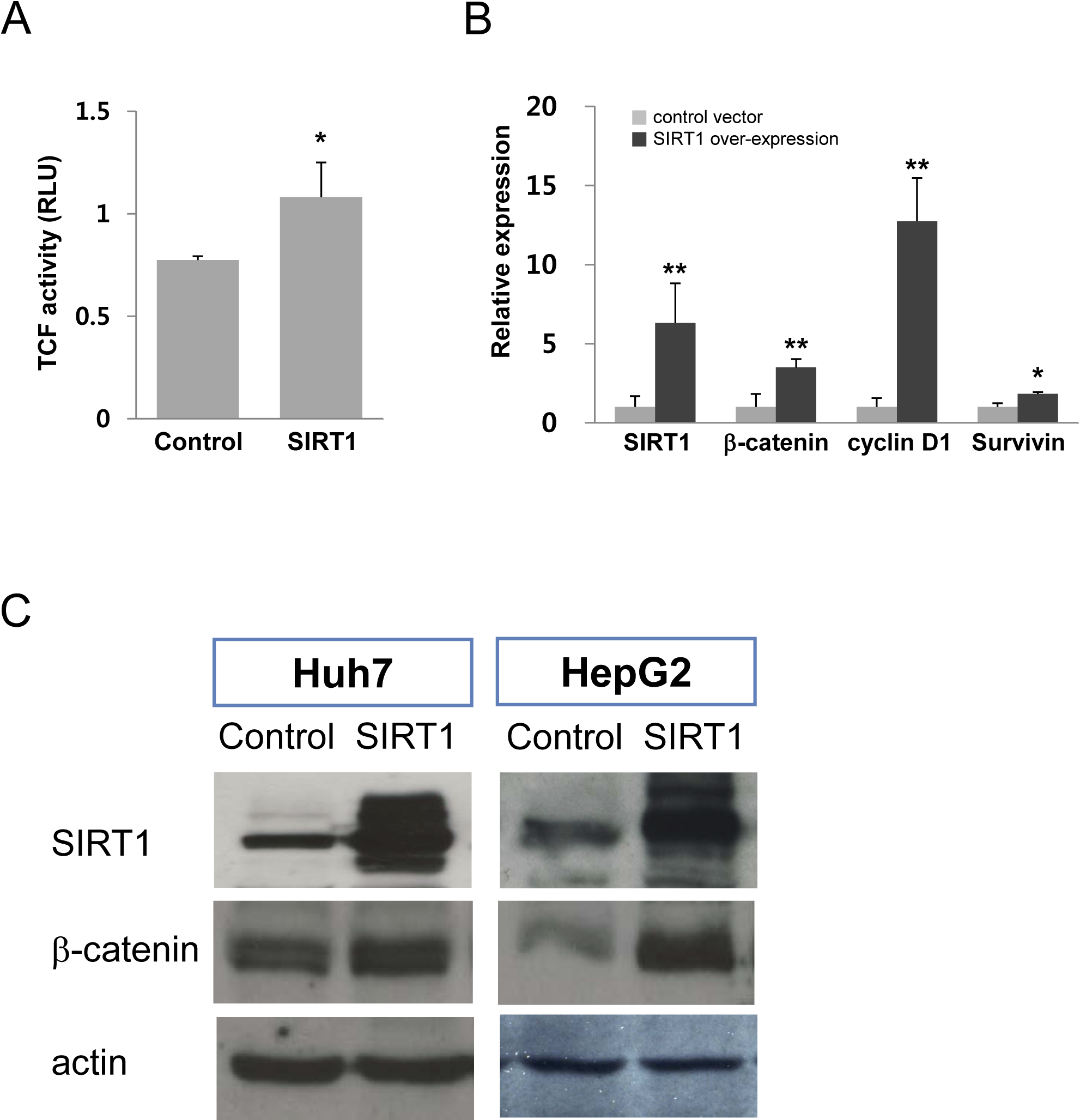

Supplement: Figure S2 — Over-expression of SIRT1 induces â-catenin expression. A) Over-expression of SIRT1 in Tet-O-MYC cells increases β-catenin/TCF reporter activity. B) Quantitative real time PCR. After transfection of Tet-O-MYC cells with a SIRT1 over-expression plasmid, the expression of mRNA for SIRT1, β-catenin, cyclin D1, and survivin significantly increases compared with control vector. C) Over-expression of SIRT1 was induced in the Huh7 and HepG2 human hepatocellular carcinoma cell lines. Over-expression of SIRT1 increases the expression of β-catenin protein. One asteriks indicate P<0.05 and two asteriks indicate P<0.001. (TIF) [file pone.0045119.s002.tif]

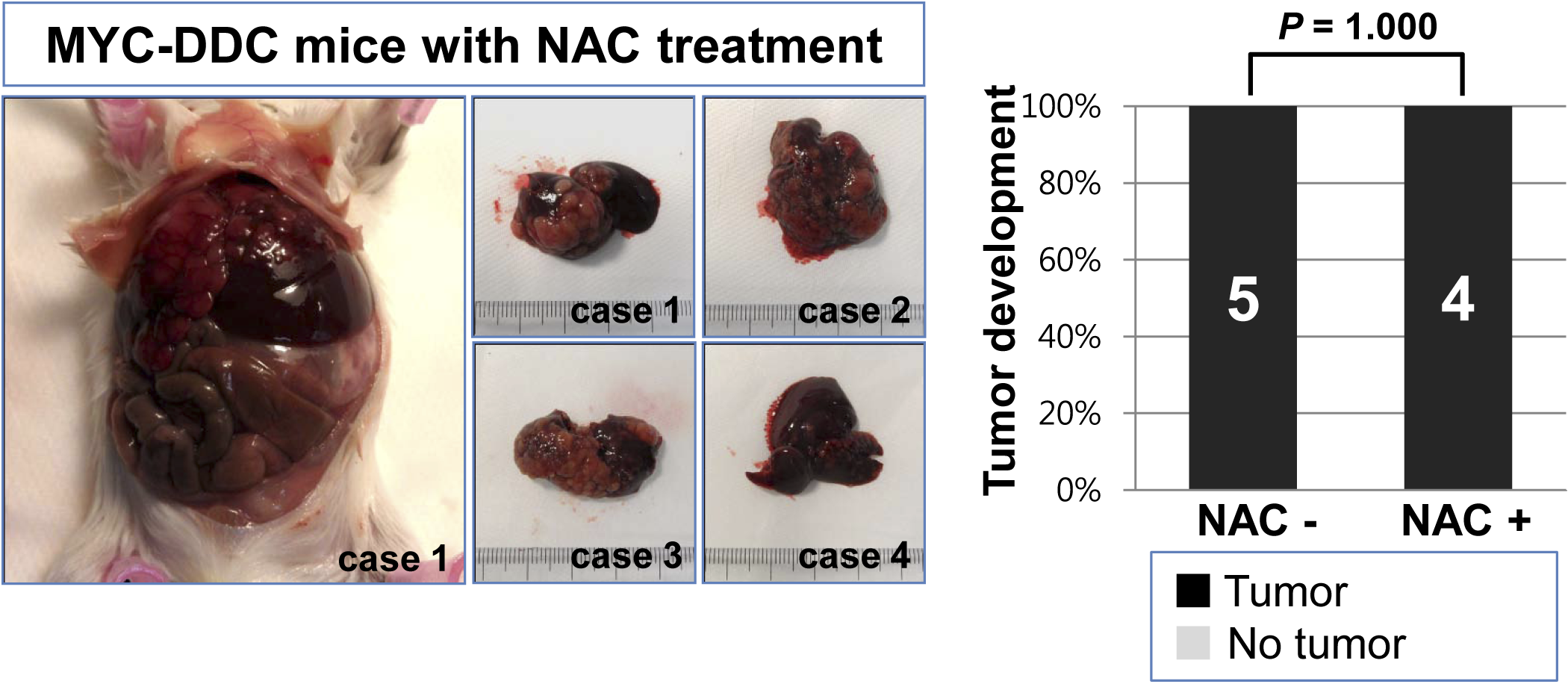

Supplement: Figure S3 — The effect of N-acetyl-L-cysteine in hepatic tumorigenesis. Administration of N-acetyl-L-cysteine (NAC) in DDC treated c-Myc transgenic mice (MYC-DDC mice) does not influence liver tumorigenesis. All four MYC-DDC mice with NAC (10 mg/mL) and all five MYC-DDC mice without NAC treatment developed hepatic tumors (P = 1.000). (TIF) [file pone.0045119.s003.tif]

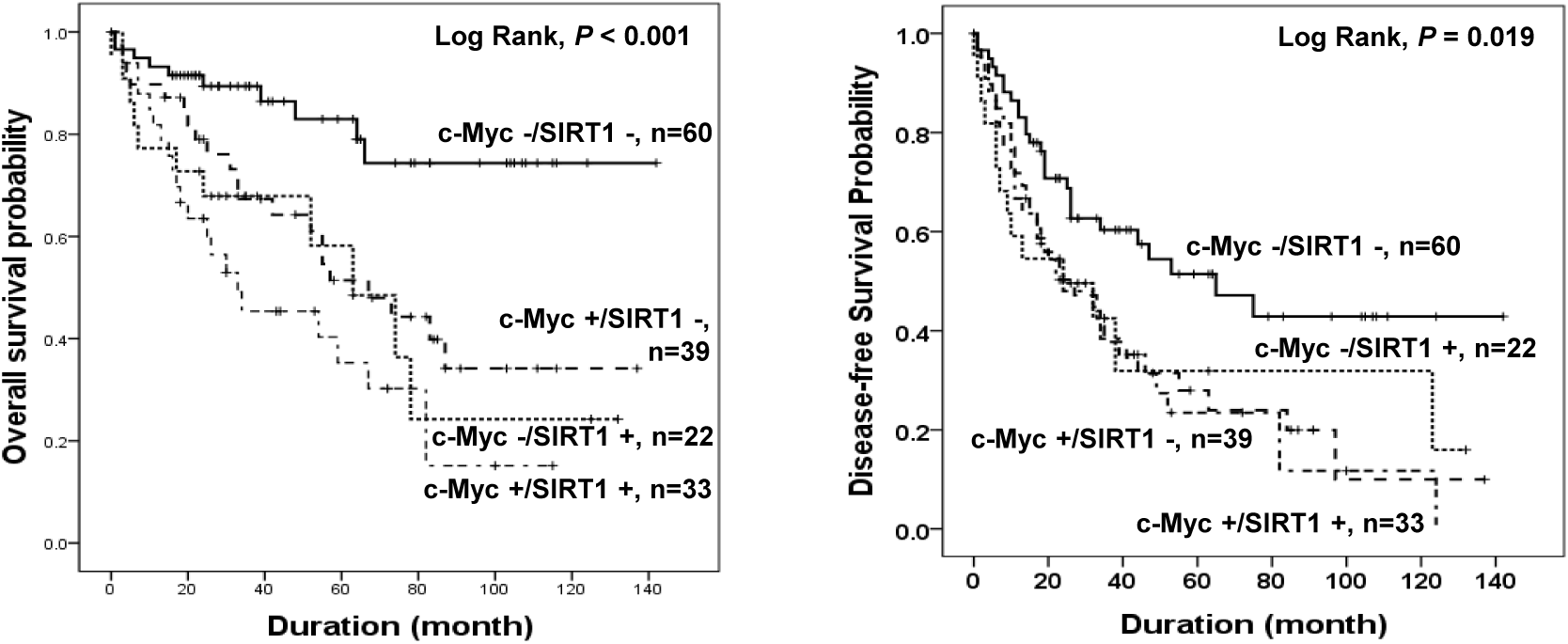

Supplement: Figure S4 — Kaplan-Meier survival analysis in hepatocellular carcinoma according to the combined expression of c-Myc and SIRT1. Overall survival and disease-free survival in 154 patients according to the combined expression of c-Myc and SIRT1 (TIF) [file pone.0045119.s004.tif]
